# Implementing a Global Health Qualitative Research Study: Experiences of a Project Coordinator in Uganda

**DOI:** 10.24248/EAHRJ-D-16-00343

**Published:** 2017-07-01

**Authors:** Jasmine Kastner, Cecilia Milford, Cecilia Akatukwasa, Annet Kembabazi, Jenni Smit

**Affiliations:** a McGill University Health Centre, Research Institute, Montreal, Canada; b University of the Witwatersrand, Faculty of Health Sciences, Durban, South Africa; c Mbarara University of Science and Technology, Mbarara, Uganda

## Abstract

Qualitative research in global health requires substantial operational and logistical support during both the implementation phase and day-to-day operations. However, little to no published work shares the experiences of international qualitative research teams. Yet, without a strong project foundation and attention to everyday details, studies can begin without appropriate guidance and, as a result, poor quality data may be generated. This paper presents a detailed account of a project coordinator's experience implementing 4 qualitative HIV and reproductive health studies in Uganda between 2012 and 2014, reflecting on our research team's practices and lessons learnt, and provides recommendations for successful project implementation. The aim of this paper is to help new global health qualitative project coordinators, and international teams more generally, by detailing 6 coordination tasks: hiring, training, team communication, organization of study documents, data collection and storage, and research ethics. To avoid repeat learning of basic, yet important, logistical steps by each new qualitative research project coordinator, this paper can help coordinators think about how to organize their work in order to prepare for both planned and unplanned challenges that have been encountered by others. Sharing operational and logistical experiences and expertise can benefit the global health community and help future studies run more efficiently.

## INTRODUCTION

While quantitative research still dominates global health, the use of qualitative research has increased over the past decade. The HIV/AIDS epidemic has been at the forefront of combining epidemiologic and social science research to address the complex mix of social, cultural, political, and economic factors related to the virus and new treatment methods.^1–10^ Powerful on its own, qualitative research has long been a crucial foundation for biomedical intervention programs, cohort studies, and clinical trials, as the success of these larger studies is context dependent.^2,11–15^ Qualitative research is essential to help identify and address the multiple realities of clinic patients and participants prior to planning and implementing interventions, programs, or large quantitative studies.

International qualitative research studies require significant operational and logistical support on the ground during both the initial set up of the research infrastructure and day-to-day operations. At times, the project coordinator position may be filled by a young professional beginning a career in global health research. Extensive studies have focused on building research capacity and optimizing the execution of a range of specific quantitative research methods in a successful and efficient manner.^16–19^ However, little information has been published on the key components of research logistics and the day-to-day operations of running a research study in the field, especially regarding qualitative research studies. The sharing of on-the-ground experiences and best practices is necessary to enhance and ensure rigour in qualitative research.

The global health community habitually emphasizes their goal to improve health for all and move towards equity for people worldwide.^20^ Qualitative research is needed to provide in-depth and contextual data about socio-behavioural issues that impact health and decision-making and to better understand new methods of improving health. Yet, little to no information is shared within the global health community about the important steps needed to conduct qualitative research during implementation of these studies. Without this knowledge, individual research groups may begin their studies without guidance or suggestions from experience gained or lessons learnt from previous studies. This can result in ad hoc qualitative research practices, which may undermine the importance of rigour in qualitative research. We believe that the research community should collectively grow from shared experiences and conversations about the common steps required to achieve quality qualitative data. For example, the methods section in most journal publications is intended to provide a replicable description of data collection and analysis procedures. Yet, the preparatory steps leading up to data collection; the logistics required to achieve and maintain high data quality, research ethics of the highest standards, and team formation, training, and participation; and overall study operations are rarely documented.

In this paper, we present a detailed account of our international team's experiences implementing 4 qualitative studies in Uganda between 2012 and 2014. We reflect on our experiences and lessons learnt, and provide recommendations for successful qualitative project implementation. This paper focuses on the 6 coordination tasks that will help guide new global health qualitative research project coordinators as they implement research studies in the field.

Our intention is to share our team's experience in order to provide first-time qualitative research coordinators with an organizational foundation that could facilitate the running of future studies smoothly. We believe that this paper will help project coordinators organize their work by being aware of and planning for planned and unplanned challenges that have been encountered before. Encouraging research teams to share their operational and logistical expertise will benefit the global health community as they build on each other's experiences. In turn, this will help researchers avoid repeat learning of the same important steps and anticipate challenges when implementing a new study.

## BACKGROUND

Our qualitative studies were designed as a follow up to quantitative study results^21,22^ and as a first step towards implementing sustainable HIV and reproductive health programs. All 4 of our global health qualitative studies were focused on HIV, reproductive health, and access to care, and were conducted in Mbarara, Uganda. Uganda has one of the highest total fertility rates globally, estimated at 6 children per woman.^23^ HIV prevalence among adults (aged 15–49 years) is estimated at 7.3%, with higher prevalence among women (8.3%) compared with men (6.1%).^24^ Our studies focused on in-depth interviews with: 1) serodiscordant couples regarding their pregnancy plans;^25^ 2) health care workers about their views and knowledge of reproductive health care for HIV patients;^26^ 3) men living with HIV about their practices and motivations around disclosure and family planning practices and; 4) recently pregnant women living with HIV and mental health care workers about postpartum depression.^27,28^ These qualitative studies were developed from an ongoing reproductive health study within the Uganda Antiretroviral Rural Treatment Outcomes (UARTO) cohort study^21^ in Mbarara, and were all separately approved under national and university research ethics boards. All participants living with HIV and their serodiscordant partners were recruited from the UARTO cohort. Health care workers were recruited by contacting clinics within the district of Mbarara.

For the purposes of this paper, the qualitative research methods used were in-depth interviews and field notes. The goal of our studies was to collect individual perspectives and lived experiences. Given the sensitivity of our research topics, our team collectively chose in-depth interviews as the most confidential and secure method for participants to share their stories. Interview guides and other supplementary information about these 4 studies can be found in the referenced publications.

Our Ugandan studies were international collaborations, which added to the complexity of the research process. Principal investigators were located in Uganda, Canada, South Africa, and the United States. The Canadian project coordinator was based in Mbarara for the duration of the studies and was supported by 3 Ugandan research assistants. The majority of team members had previous experiences implementing studies in Mbarara, Uganda.

**FIGURE 1.**
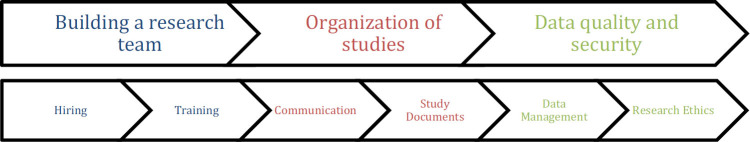
Experience and Advice on Key Components to Study Logistics

In the following sections, we share our team's experience by outlining 6 important tasks for implementing qualitative research studies in global health with a multi-national team. In each section, we break down our experience into implementation, lessons learnt, and recommendations for best practice (Figure 1).

## BUILDING A RESEARCH TEAM

### Hiring Field Staff

#### Implementation

A research job advertisement in Uganda, where unemployment rates for the under-30 working population is 35%,^29^ receives many applicants. Our interview committee consisted of the Ugandan principal investigator, the project coordinator, the human resource manager, and a second project coordinator. Having a diverse interview committee helped us conduct thorough interviews with the candidates, and rank each candidate's interview responses and work experience.

Our interview committee was equipped with a list of questions for all candidates that covered qualitative research experience, general work experience, work ethic, the understanding and ability to implement the principles of research ethics, personal expectations, and personal interest in the research topic and method. We also explored their personal values and level of comfort talking about the sexual and reproductive health issues we were researching. These questions helped us to assess if a candidate would be a good match for our research team.

At the end of each interview, the candidate was asked to conduct a mock interview with 1 person on our interview committee. This mock interview assessed the candidate's comfort conducting an interview focused on HIV and reproductive health, and his/her fluency in the language in which the study's interviews were to be conducted. The combination of the prepared questions and the mock interview helped the interview committee evaluate a best-fit candidate for the research team.

#### Lessons Learnt

Although conducting a mock interview with each candidate was time consuming, this method of assessing the applicant's qualifications was indispensable. Through these interviews, we learnt that while not all eligible candidates may have advanced degrees or experience in qualitative research, consideration should also be given to candidates who show natural interview skills and a personality that is compatible with the research team. During fieldwork, we also learnt that hiring research assistants with similar demographics as the potential participants – such as age, sex, tribe/ethnic group, and community – helps make participants feel comfortable to open up and share their stories during interviews. Our team also took on a student trainee who benefited from the training and was later hired as a full-time research assistant.

#### Recommendations for Best Practice

We recommend including bi-annual evaluations in an employee's contract. These coordinator evaluations ensure formal feedback is prepared about the employee for both the principal investigators and employee her/himself. The bi-annual evaluation should be formally written and discussed in a meeting between the project coordinator and research assistant, as well as by a conference call with the international principal investigators. The evaluation should focus on the research assistant's successes and areas for improvement. The formal feedback mechanism is appreciated and seen as a good time to discuss career goals shared during the hiring process and a way to guide professional development. It also serves as a platform to address any problems and areas to continue working on. This process enables the project coordinator to facilitate career advancement when possible; an essential part to increasing research capacity at the field site.

### Training

#### Implementation

Over the course of a study, staff training is a continuous process, with the bulk of training concentrated at the beginning of a project. While the initial training for each study was time consuming, it contributed to the study's success. Our project coordinator spent 2 weeks of full-time training to familiarise the team – 3 new research assistants and 1 student – on the study goals and methods. The initial training focused on the differences between qualitative and quantitative research, background of qualitative research, different interview techniques, body language during interviews, research ethics and maintaining confidentiality (including informed consent and interview protocol), conducting practice interviews, standard operating procedures, and an introduction to data analysis. Training information was provided to research assistants through oral presentations, PowerPoint slides, research articles, training articles, and fact sheets. Even though some research assistants had previous qualitative research experience, all team members agreed that the basic training helped them achieve a common skill level, set values, and purpose to the studies. It was also a useful way for the project coordinator and the research assistants to get to know each other.

Once the initial training on general qualitative research methods was complete, the project coordinator built on the qualitative skills recently learnt by introducing the protocol of the first study. This enabled all staff to understand the purpose of the study, previous work that had been completed in the area of research, and approach to be used to conduct the study. We then moved on to review the interview guide and conduct mock interviews with the research assistants. The mock interviews provided research assistants an opportunity to become comfortable with the study interview guide and understand the purpose of each question, while practicing their qualitative interview skills.

#### Lessons Learnt

Consistent with previous research,^30,31^ we found that training in a small group created a productive learning environment. Peer review and feedback were also an essential part of our training method. These activities gave Ugandan research assistants the opportunity to learn from each other and provide invaluable cultural insight and suggestions for changes to the study documents. These suggestions improved the interview guide and made for more successful interviews, something that would have been lost to non-local staff.

Practising with the study's interview guide also provided the project coordinator an opportunity to understand research assistants’ level of knowledge about the health background of the study – in our case, HIV, reproductive health, and mental health. We learnt that if research assistants were not comfortable explaining the study purpose or responding to basic questions about the research topic to peers, the actual study interviews lacked the in-depth content desired. Moreover, understanding the purpose of why each question is included in the interview guide was imperative, as was allowing the research assistant to expand or rephrase questions during an interview so that a participant could share their story. Secondary training was conducted by the project coordinator whenever a research assistant struggled to explain the purpose of the study or a particular question. When necessary, the purpose and background of the study was reviewed. Our team prepared multiple short trainings on HIV, family planning, and mental health over the span of the projects. These trainings were critical to the success of our qualitative studies since they instilled confidence in the research assistants when conducting the interviews and facilitated understanding of when and where to probe deeper on participant responses during interviews.

Although an intense initial training program did lead to an initial time lag in data collection, the time dedicated to training and preparation was more than compensated by structured data collection and good quality data. Training research assistants on interview techniques and protocol should not be limited to the beginning of the study, it is a process that should continue throughout the duration of a study. We learnt that feedback from the project coordinator and principal investigators after the first few interviews of a study helped highlight any persistent misunderstandings about certain questions in the interview questionnaire. Providing constructive criticism was often welcomed as a way to improve personal interview skills and the overall quality of the study. We also learnt that these feedback conversations created a comfortable space for research assistants to express any struggles they may have with asking certain questions or with the flow of the interview guide.

#### Recommendations for Best Practice

We recommend that feedback to research assistants should include a review of the purpose of the study, relevance of each interview question, research ethics protocol, and suggestions on how to improve probing during participant interviews. The training and review processes listed in the above section help to ensure that research and ethics protocols are maintained while improving the quality of the study data collected. We conducted regular protocol reviews with the research assistants in order to keep the team up to date. A training and review process can create a positive feedback loop that benefits both research assistants and the study findings. We noted that with better information, the data improved, and, subsequently, the confidence and skills of the research assistants also improved.

Feedback from research assistants during the training periods and initial data collection can help improve the quality of the study through small modifications to the interview guide and study protocol, ensuring that studies are optimized for the respective cultural framework(s). Such small modifications may include revising the wording of interview questions to address social and cultural norms, which may require research ethics amendments, and enable better flow of the interview guide, more thorough responses from participants, more appropriate translation of study documents, and improvement of data quality. We submitted all interview guide modifications to our respective research ethics board (REB). Uniformity across all interviews remained since participant interviews began only after the initial training period – where modification to study documents were made – and after REB amendments were approved.

## ORGANIZATION OF STUDIES

### Communication

#### Implementation

Team communication is important for any study. Since our qualitative research team was small (less than 10 people in total), the project coordinator, 3 research assistants, and 5 principal investigators scheduled regular meetings to discuss how the interviews were progressing and to anticipate any possible upcoming difficulties. It was essential for our local team to remain in daily contact in order to make sure the interviews were up to our standards and discuss any logistical problems. Day-to-day communication was conducted with field site staff – 1 project coordinator, 3 research assistants, and 2 principal investigators – both in person and via email or Skype when including the 3 international principal investigators. Beyond the local team meetings, our project coordinator and international principal investigators met weekly over Skype to discuss the progress of the studies.

#### Lessons Learnt

Commitment to scheduling and preparing team meetings required flexibility since we were based across multiple time zones. Coordinating meetings between Uganda, Canada, South Africa, and the United States required some staff to participate outside normal working hours, meeting agendas to be circulated early, and access to well-functioning communication networks. Occasionally, online meetings had to be re-scheduled or changed to email or phone conversations when the internet in Uganda was not reliable. It was the role of the project coordinator to organize team meetings and ensure that all relevant documents were circulated beforehand.

Having regular team meetings – both via telecommunication and in-person – helps integrate all research members into the study and resolve potential challenges efficiently. Team meetings also provide accountability checkpoints, since all members may be assigned work tasks. Team member progression on these tasks can be recorded in the meeting notes, circulated to all, and discussed at the start of the following meeting. In our project, knowing that all team members were working on a study task, helped boost individual work levels, as everyone understood their role in the team and responsibility in the team effort.

#### Recommendations for Best Practice

As a study progresses, it is beneficial to encourage research assistants or younger team members to lead team calls or meetings and provide feedback on how fieldwork is progressing. The importance of research assistants leading calls or meetings is aligned with the bilateral training and review process (see the Study Documents section). In a small qualitative research team, these steps empower research assistants or other team members to help develop the study rather than just follow assigned tasks. Active participation in study calls or meetings also means that all study members have an understanding of the larger framework and purposes of the study – that they are not just performing activities in isolation for the bigger project. In our study, interactive communication – through sending emails to principal investigators, sharing opinions during meetings, and asking for clarification – enabled research assistants to develop a sense of ownership in the work they were doing while promoting capacity building.

Finally, we recommend informal conversations amongst onsite staff about the progress of interviews and debriefs after each interview. Such conversations are also opportunities to provide reminders about training points, help research assistants improve interviews, and debrief about difficult interviews. In our study, these conversations were initiated by the project coordinator, with respective research assistants, and facilitated the operation of each study.

### Study Documents

#### Implementation

Beyond study protocols, research ethics documents, interview guides, and other general study documents, we found 3 sets of documents to be essential to the success of our qualitative research studies. These 3 document sets included: standard operating procedures (SOPs), interview recruitment scripts, and memorandums of understanding (MOU). While commonly used for quantitative studies, these documents are not often required for qualitative studies. However, we believe they should be part of project implementation. The documents were developed by the project coordinator during the initial phase of our studies, outlining all study procedures in detail. All of these documents ensured that our team members were following the same guidelines and study protocols, and facilitated smooth study operations, and could be referenced when needed.

It was important to design protocols and SOPs that described all steps of our studies. These documents outlined operational procedures and were the primary reference source for future interest in replicating our studies. More importantly, the SOPs were used throughout each of our studies to set a general standard for all logistics. SOPs helped reinforce that study procedures were implemented in a uniform fashion, and ensure data collection practices met our high standards.

The SOPs were a resource for all study members. They described in detail how to schedule an interview, what documents and equipment were required for each interview, what was considered best interview etiquette, how to ensure confidentiality for participants, what transcription procedures should be used, where to record and store all data securely after each interview, where to record study expenses, and where all documents and supplies should be kept. All study procedures were written by the project coordinator and circulated to team members for review and input before being finalized. SOPs were circulated to all onsite team members in print and uploaded to the study cloud storage space for all to access as needed (more on this in the Data Quality and Security-Implementation section).

#### Lessons Learnt

After reviewing early participant recruitment notes and questions posed by participants during the interview, we learnt that it was important for research assistants to have a clear and consistent script to introduce the study to eligible potential participants. These scripts were available in the local language and could be used for in-person or phone conversations.

The short scripts were used to guide the research assistants during participant recruitment and were useful tools to guarantee uniform recruitment and a clear presentation of the study purpose and details to eligible participants. They also ensured that all potential participants were aware that the study would include an interview; that they would be compensated for their time; and that we would call the day before their scheduled interview day to confirm their participation. We also learnt that this last point was important for planning our daily work schedules and minimizing unnecessary money spent in the study budget for transport.

SOPs and recruitment scripts were particularly useful during the early recruitment phase when research assistants were still getting used to the study. These documents helped to guarantee that all important details were shared with eligible participants. As the studies progressed, SOPs and scripts were phased out and only used when necessary. All study protocols and scripts were reviewed and shared with team members.

#### Recommendations for Best Practice

Finally, when principal investigators are located across many countries, we recommend that an MOU between each investigator be written. An MOU describes the expectations and specific roles of each principal investigator during each phase of the study. It is ideal to create and circulate this document during the early planning stage of a study. This ensures that all principal investigators agree on their respective workload during the study design, implementation, analysis, write-up, and dissemination of the study. Once agreed upon, an MOU is a contract to help ensure that the study runs smoothly and each designated principal investigator completes their assigned section of the study on time.

## DATA QUALITY AND SECURITY

### Data Management

#### Implementation

This section will explain the procedure we followed after each interview was conducted. Although qualitative research differs from clinical trials and quantitative research, research ethics guidelines and good clinical practices must be followed to protect participants and ensure good-quality research. Our team made the decision that only 1 interview per research assistant could be completed per day. This assured uniformity across all interviews. The rest of the day was spent preparing for the interview, completing field notes after the interview, meeting with the coordinator to discuss how the interview went, and beginning the transcription process.

The project coordinator prioritized safe storage of all interview data. Documenting demographic data and creating summaries after each interview, rather than logging data at the end of the study, helped our team with discussions about the progress of our studies and the identification of interesting findings. This helped us to prepare for conference paper submissions, write progress reports, and present informal findings to our research collaborators. Furthermore, by updating study data and information after each interview, we were able to identify any mistakes and resolve any queries with the participant, timely and with accuracy.

After each interview, the research assistant who conducted the interview added information to 3 ongoing spreadsheets shared with all team members: demographic data collected at the start of the interview; interview details, such as location of the interview and expenses incurred; and short interview summaries. Collecting a brief summary of each interview in a single document was useful during the analysis phase of the study, as it provided a quick overview of the main points of an interview and saved time that would have been spent reading lengthy transcripts.

The next step in the data collection process was for the research assistant to transcribe and translate the new interview from the local language to English. This was done using Express Scribe software and following the relevant SOP. Once complete, a different research assistant reviewed the interview transcript while listening to the audio. This process provided an opportunity to clarify translation errors, catch phrases that were missed during transcription, and correct misspellings. The final review was completed by the project coordinator and, sometimes, a principal investigator to clarify any cultural concepts or questions in the transcript.

#### Lessons Learnt

It is important to note that transcription and translation activities are time consuming for all staff. One hour of audio can take from 6 to 8 hours to be translated and transcribed. The transcription review by a separate research assistant typically takes 2 to 3 times the length of the interview, as does the final review. Additional time for feedback from each interview must also be added, especially during the early stages of a study. Given this, an average of 1 to 2 interviews per week per research assistant was our norm, depending on the length of the interview. Our studies did not conduct any focus groups, but it is important to note that the transcription of focus groups often takes longer than one-onone interviews.

Research assistants were encouraged to add field notes at the end of the interview transcription. Writing field notes is standard protocol in qualitative research^32–36^ and provides an opportunity for the research assistant to explain how the participant was acting, what body language was used, and any additional information not available from the audio recording. Along with the short interview summaries in the shared spreadsheet, we found that field notes often helped our team understand the interviews better since they summarized the interview and included additional comments from the research assistant who conducted the interview.

All study documents were secured with standardized file names, for identification ease; audio files and password-protected transcripts and spreadsheets were stored on an encrypted and password-protected virtual cloud. This secure virtual cloud allowed for real-time access to all authorized team members. We learnt that having 1 person responsible for storing and organizing study documents helps to avoid confusion. Our project coordinator was in charge of keeping all study documents up-to-date and all study folders organized on the virtual cloud and in hardcopy format. This role included ensuring that the most recent SOPs, study budgets, progress reports, interview guides, transcripts, summaries, and demographic data were uploaded online. Signed consent forms were stored as hardcopy documents only, and all hardcopy study documents and equipment were stored in a key-locked filing cabinet in a secure research office.

#### Recommendations for Best Practice

Our project coordinator's role was to ensure that all interview information – such as an identification number associated with the person interviewed, location and time of interview, and transport refunded – was entered into the online spreadsheet timely and accurately; to conduct a final review of all transcripts; and to ensure demographic data for each participant was up-to-date. We recommend that a designated staff member be responsible for removing the audio file from the voice recorder and storing it in a protected and safe backup location. Once the interview transcript has been reviewed and queries clarified, the electronic transcript can be stored with its respective audio file for future reference.

Following good clinical practice guidelines, labelling and organizing hard copy storage of all research ethics documents will save time when writing REB renewals or searching for study data. We recommend assigning separate files for storing signed informed consent documents, research ethics documents, SOPs, scripts, and interview guides. Organizing hardcopy files is useful when documents have to be shared with other research collaboration team members, when there is need to consult original participant documents, or when it is necessary to prepare for an audit.

### Research Ethics

#### Implementation

While research ethics falls under the ‘data quality and security’ heading, this theme cross-cuts all sections of this paper due to its importance throughout the entire research process: from preparing study documents, screening participants, recruitment, consent, interviewing, and data collection and analysis, to the dissemination of findings. The principal purpose of research ethics is to ensure the participant's rights are not violated in any way, and that confidentiality is maintained. In qualitative research studies, research assistants often conduct interviews about personal and sensitive topics – in our case, HIV, sexual practices, depression, partner relationships, and pregnancy. All of our interviewers were trained – through online and in-person ethics and good clinical practice workshops – in maintaining research ethics and protecting the rights of the participants that may arise in any interview situation. Training was also provided to ensure the research assistants conducting interviews were comfortable pausing or stopping an interview if a participant was uncomfortable, not ready to continue, and distressed; if they needed to be referred for counselling or treatment; or if someone interrupted the interview. Most interviews were conducted in a private room adjacent to the HIV clinic, with only a few participants electing to conduct the interview in the privacy of their own home.

#### Lessons Learnt

We learnt that participants in our studies sometimes assumed that the research assistant conducting the interview was a health care provider and sought advice from them. This occurred despite the fact that during study introduction and the consent process research assistants stated that they were not providers. The project coordinator provided research assistants with training on how to avoid feeling as though providing health advice may be the only way to move forward with the interview. While it was necessary for research assistants to understand the health background of the study in order to conduct thorough interviews, it was equally as important for the research assistant to understand that their role was to conduct a thorough interview and not to provide health advice they were not professionally trained to give.

After the first encounter of this situation, the project coordinator designed a health care referral form and SOP for interviewers to use if their participants asked for health advice. The SOP included a script for explaining that the interviewers are not health care professionals but could refer the participant to a health care professional after the interview. The referral form included the name and phone number of health care professionals from HIV and mental health clinics who had volunteered to speak with any participants if they had further health questions.

#### Recommendations for Best Practice

Beyond maintaining research ethics before, during, and after interviews, it is the role of a project coordinator to apply for research ethics renewal and request approval for amendments to all studies. Missing an REB deadline can force a study to pause data collection while waiting for REB renewal approval, resulting in a loss of time, money, and the pace of the study. Having a multi-national collaboration required our project coordinator to keep track of research ethics approvals and renewal dates for 4 different studies across 3 different countries. We recommend that the project coordinator create 1 document and 1 calendar that lists all research ethics committee approvals and expiry dates to ensure timely research ethics renewal applications amidst other operational tasks.

Since our research team was based within a larger research collaboration, we received help with submitting our application packages to the 4 institutions for which our studies required REB approval. The advice and help received from our colleagues was invaluable in navigating international research board applications, amendments, and renewals. We recommend that first-time project coordinators seek advice from colleagues with experience. Knowledgeable colleagues can share research ethics cover letters and application forms, and provide insightful tips for submitting REB application packages correctly. They can also advise on the estimated time for study feedback on application submissions, thus helping with appropriate study planning.

## DISCUSSION

**FIGURE 2.**
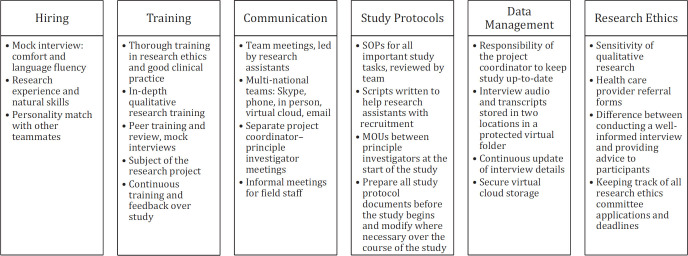
Summary of Key Points

Abbreviations: MOU, memorandum of understanding; SOP, statement of purpose

Interest in qualitative research in global health is growing. Over the course of our studies presented in this paper, many of our research collaboration team members were excited to learn about our work and expressed interest in learning more about the qualitative research methods used in our studies and the lessons shared in this paper. The points presented are a combination of lessons learnt and recommendations for project coordinators to manage operations and ensure their future projects move forward smoothly while assuring data quality. These points are useful references for both first-time and experienced qualitative research project coordinators in global health. Any new international qualitative research team member can benefit from learning from the shared experiences of other researchers (Figure 2).

While each of the key points are presented separately, they are interrelated and build on each other. Beyond what is shared in this paper, we recommend that a new project coordinator seek out a mentor at the field site to provide advice about the research collaboration – a group of researchers from different organizations working together in a similar field of research, sharing data and ideas – including expected timelines to complete requisite responsibilities, how REB application(s) should be prepared, who to contact for help with various work tasks, samples of previous work, and other general support. Collaborating with colleagues and peers is also a useful way to learn about the most efficient techniques to manage various coordinator duties that are common across all studies at a field site. Collaborating with other project coordinators at the field site also promotes an open environment to ask for help when challenges arise.

Our team valued the assistance of our collaboration team members, the health clinic staff, and participants. Before beginning each study, we introduced the purpose of the study and methods used to clinic and research collaboration staff members so that all were aware of the work we were doing. We also disseminated our preliminary research findings at the end of the data collection phase to the collaboration and clinic staff in order to share the findings of our studies and maintain a good relationship. Both introductory and dissemination presentations are an important part of collaboration and conducting research. In doing so, a shared learning environment was created and our colleagues were helpful in moving the studies forward in order to learn more about HIV and reproductive health in the region.

In addition to the above, the Canadian project coordinator living in Uganda made it a priority to focus on equitable practices, which entailed open conversations about both work and social life, being open to sharing problems and asking for advice or help from colleagues, sharing a same small workspace with all team members, and attending research collaboration and clinic meetings. There were times where the international project coordinator was put into a position of power,^37,38^ yet when this occurred, navigating both international and local staff status was simplified through discussion about the tasks to be completed and the job roles of each person involved. Hard work and sharing the weight of tasks fairly was a way to avoid dogmatic views of international and local staff barriers within global health research. By constantly being aware of unspoken norms of power dynamics and privilege in global health research, the project coordinator focused on actively being part of the research and clinic teams by learning from, listening, and adapting to colleagues. Without the above-mentioned points in this discussion, our studies would not have been completed as efficiently or as successfully. Collaboration with other research teams, awareness of power dynamics, and sharing our study details with non-research colleagues created a productive work environment.

Finally, the operational and study logistic key points described in this paper are based exclusively on our qualitative research field site experience. Certain aspects of these points, like research ethics and study documents, can overlap with operational procedures of quantitative or mixed-method research. Yet, it is the combination of operational procedures across all 6 key components (Figure 2) that makes the operational tasks described in this paper unique to implementing a qualitative research study. It is also important to state that this paper focused on the data collection phase of qualitative research and did not delve into the rigorous process of data analysis to ensure results are interpreted correctly, or that they are reliable and valid.

## CONCLUSION

Understanding your research collaboration's team dynamics, in combination with good interpersonal skills, is an important early step for any new project coordinator. Many of the key points explained in this paper can be completed through a ‘learning-by-doing’ process if project coordinators are provided with good mentors and peers for guidance. Reading about the experience and lessons learnt from previous studies is resourceful preparation for an upcoming project.

This paper hopes to promote communication within the global health qualitative research community. It is intended to help new project coordinators by detailing 6 key points that we believe help an international study team prepare for planned and unplanned challenges encountered by others in the past and assure data quality, while maintaining rigour in qualitative research.
